# Hyperuricemia in psoriatic arthritis: clinical correlations and implications

**DOI:** 10.1007/s10067-025-07400-4

**Published:** 2025-04-07

**Authors:** Samar AbdAlhamed Tabra, Hany M. Aly, Saad Ghanem, Mohammed Hassan Abu-Zaid

**Affiliations:** 1 Department of Rheumatology and Rehabilitation, Faculty of Medicine, Tanta University, El-Geish Street, Tanta, 31527 Gharbia Egypt; 2 Rheumatology and Rehabilitation Department, Faculty of Medicine, Al-Azhar University, Cairo, Egypt; 3 Rheumatology and Rehabilitation Department, Faculty of Medicine, Al-Azhar University, Damietta, Egypt

**Keywords:** Comorbidity, Disease activity, Hyperuricemia, Psoriatic arthritis

## Abstract

**Background:**

Psoriatic arthritis (PsA) patients may have elevated serum uric acid levels, and hyperuricemia may impact the degree of inflammation and clinical disease severity.

**Objectives:**

comparison between PsA patients with and without hyperuricemia and assessment of the effect of hyperuricemia on clinical presentation, disease activity, disease severity, and associated comorbidities in PsA patients.

**Methods:**

76 PsA patients with hyperuricemia and 74 PsA patients with normal uric acid as control were included. Demographic, clinical, comorbidities, and laboratory data were collected. Hyperuricemia threshold ≥ 60 mg/L.

**Results:**

There were no significant differences between patients with and without hyperuricemia regarding gender, PsA articular subtype, PASI score, and treatment received, while patients with hyperuricemia were older (40.47 ± 8.53 vs 34.59 ± 7.29, *p* = 0.0001), had more comorbidity, higher body mass index (BMI) (28.49 ± 2.07 vs 26.91 ± 1.63kg/m2, *p* = 0.0001), DAPSA score (16.75 ± 7.04 vs 9.32 ± 6.35, *p* = 0.0001), ESR (34.78 ± 7.12 vs 28.55 ± 8.97, *p* = 0.0001), CRP (11.42 ± 3.23 vs 8.68 ± 4.04, *p* = 0.0001), serum cholesterol (220.42 ± 46.83 vs 169.82 ± 37.82, *p* = 0.0001), and triglycerides (136.47 ± 36.4 vs 104.89 ± 22.15, *p* = 0.0001), and longer duration of Psoriasis and PsA. The serum uric acid levels were significantly positively correlated with age, duration of Psoriasis, duration of PsA, BMI, CRP, ESR, DAPSA, and PASI score. Multivariate analysis showed that male sex, BMI, and increased disease activity were independent predictors of hyperuricemia in PsA patients.

**Conclusion:**

Psoriatic arthritis patients with hyperuricemia have higher age, BMI, disease activity, and more associated comorbidities. In PsA patients, hyperuricemia was associated with male sex, BMI, and increased disease activity, but not associated with PASI score.

**Table:** 

**Key Points** • *Psoriasis, PsA, and hyperurice s is a bi-centric case–control retrospective of cardiovascular disease.* • *Male gender, BMI, and increased disease activity were independent predictors of hyperuricemia in PsA patients.* *• Psoriatic arthritis patients with hyperuricemia have been more associated with comorbidities.*

**Supplementary Information:**

The online version contains supplementary material available at 10.1007/s10067-025-07400-4.

## Introduction

Psoriatic arthritis (PsA) is an inflammatory arthritis associated with psoriasis and affects 20–30% of patients with psoriasis [1].

Psoriatic arthritis and gout are two common diseases that can occur simultaneously. The pathophysiological mechanisms of the link between these two disorders are still unknown [2].

Psoriasis, PsA, and hyperuricemia are recognized to be associated with metabolic syndrome and a higher risk of cardiovascular disease [3].

According to previous research studies, a significant correlation was reported between serum uric acid and psoriasis severity [4]. Then, other research has shown contradictory findings [5].

Few observational studies evaluate the effect of hyperuricemia and/or gout on PsA, and its impact on clinical presentation, disease activity, and severity [3, 6–8].

This study aims to compare the hyperuricemic and normouricemic PsA patients and to assess the effect of hyperuricemia on clinical presentation, disease activity, disease severity, and associated comorbidities in PsA patients.

## Materials and methods

This is a bi-centric case–control retrospective study.

### Setting

Patients were selected from the outpatient clinic of Rheumatology and Rehabilitation departments, Tanta & Al Azhar University Hospitals.

### Patients

150 Patients meeting CASPAR [9] criteria for PsA (76 PsA patients with hyperuricemia and 74 PsA patients with normouricemia as control) were included in this study, the data were collected from Tanta University hospital, and Al Azhar University hospital.

Hyperuricemia threshold ≥ 360 μmol/L was used to compare patients with normo-uricemia and hyperuricemia. The 2015 ACR/EULAR criteria for gout indicate this threshold of 360 μmol/L (60 mg/L) [10, 11].To reduce the impact of a single, isolated hyperuricemia event, we calculated each patient’s median value of serum uric acid (of at least 4 visits through the last 12 months) to see if they were above this cutoff value.

Patients with other dermatological disorders or another autoimmune disease other than PsA were excluded. Also, we excluded patients who had acute renal failure at the time of the serum uric acid measurement, those who had hyperuricemia based on a single serum uric acid test, clear gouty patients according to the 2015 ACR/EULAR criteria for gout, and patients taking medication that affects serum uric acid levels such as thiazide, ethambutol cyclosporin, and pyrazinamide.

### Ethics approval and consent to participate

This study is in agreement with the ethical guidelines of the Declaration of Helsinki, and it follows the ethical standards of Tanta Faculty of Medicine, with the institution’s ethics board approval number 36264PR203/5/23.

A complete history, physical examination, and laboratory assessment were collected and the mean value of at least 4 visits through the last 12 months was calculated.

### Clinical assessment

Demographic data included patient’s age, sex, duration of PSO and PsA, PsA subtype, body mass index (BMI), postmenopausal status, comorbidities (cardiovascular "hypertension: diastolic > 90 and/or systolic > 140, ischemic heart disease", hypercholesterolemia (> 200 mg/dL), hypertriglyceridemia (> 200 mg/dL), diabetes mellitus, and renal stones), and smoking status.

PSA activity using Disease Activity Index for Psoriatic Arthritis (DAPSA) scores [12] were collected from patients’ files. DAPSA was measured using tender and swollen joint counts, patient pain and patient global assessments, and acute phase reactants. The mean DAPSA from the 4 patient visits through the last 12 months was calculated.

The severity and extent of psoriasis were assessed by the Psoriasis Area and Severity Index (PASI) score; a representative of the area of psoriasis is selected for each body region. The intensity of redness, thickness, and scaling of the psoriasis is assessed as none (0), mild (1), moderate (2), severe (3), or very severe (4) [13]. The mean PASI from the 4 patient visits through the last 12 months was calculated.

### Laboratory assessment

- Routine laboratory assessment: CRP, ESR, cholesterol, and triglyceride levels.
- Serum uric acid measurements “during the last year” for each patient were reported to calculate the median of serum uric acid level for each patient.

### Statistical analysis

SPSS version 20 was used for statistical analysis of the data [14]. Numbers and percentages were used to describe the qualitative data. The Kolmogorov–Smirnov& Shapiro–Wilk test was used to verify the normality of distribution. The mean and standard deviation were used to characterize the quantitative data. The categorical data were compared using the chi-square test. Student *t*-test was used to compare two groups for normally distributed quantitative variables. Correlation between variables was done by Pearson’s correlation coefficient. For evaluation of the factors associated with hyperuricemia univariate and multivariate analyses were done (only covariates showing a p-value < 0.05 at univariate analysis were included in the multivariate logistic regression analysis). P value less than 0.05 was considered statistically significant.

## Results

One hundred and fifty Patients meeting CASPAR criteria for PsA (76 PsA patients with hyperuricemia and 74 PsA patients with normouricemia) were included in this study. There were no significant differences between hyperuricemic and normouricemic patients regarding gender, PsA articular subtype, PASI score, and treatment received, while hyperuricemic PsA patients were older, had more comorbidity, higher BMI, DAPSA score, ESR, CRP, serum cholesterol, and triglycerides, and longer duration of PsO and PsA. The demographic data, disease-related characteristics of the patients, and laboratory findings are shown on Tables 1 and 2.

**Table 1:** Demographic and disease-related characteristics of the patients

	Hyperuricemic PsA patients (76)	Normouricemic PsA patients (74)	*P* value
	Mean±SD	Mean±SD	
Age (years)	40.47±8.53	34.59±7.29	0.0001*
Gender: (male/female)	32/44	28/46	0.59
BMI	28.49±2.07	26.91±1.63	0.0001*
Duration of Pso (months)	93.37±37.68	59.89±27.09	0.0001*
Duration of PsA (months)	46.11±31.29	22.67±17.38	0.0001*
Smoking: (yes/no)	23/53	20/54	0.66
Comorbidities (yes/no)	48/28	15/59	< 0.0001*
PsA articular subtype: (N of patients)			
• polyarticular: • oligoarticular: • axial: • arthritis mutilans:	26 39 11 0	24 40 10 0	0.9
DAPSA	16.75±7.04	9.32±6.35	˂0.0001*
DAPSA: (N of patients)			
• Remission: • Low disease activity: • Moderate disease activity: • High disease activity:	7 26 39 4	13 36 23 2	0.04*
PASI score	3.33±2.75	2.54±2.28	0.057
Treatment received:			
csDMARDs bDMARDs Combined csDMARDs & bDMARDs	34 5 37	27 7 40	0.54

*BMI* body mass index,* Pso *psoriasis, *PsA* psoriatic arthritis, *N *number, *DAPSA Disease Activity Index for Psoriatic Arthritis, PASI *Psoriasis Area and Severity Index,* csDMARDs* convential disease modifying anti-rheumatic drugs,* bDMARDs* biological disease modifying anti-rheumatic drugs

Significant *p* value if <0.05*

**Table 2:** Laboratory characteristics of patients

	Hyperuricemic PsA patients (76)	Normouricemic PsA patients (74)	*P* value
	Mean±SD	Mean±SD	
Serum uric acid (mg/dl)	7.27±0.66	4.62±0.7	0.0001*
ESR (first hour)	34.78±7.12	28.55±8.97	0.0001*
CRP (mg/dl)	11.42±3.23	8.68±4.04	0.0001*
Seum cholesterol (mg/dl)	220.42±46.83	169.82±37.82	0.0001*
Serum triglyceride: (mg/dl)	136.47±36.4	104.89±22.15	0.0001*

*ESR* erythrocyte sedimentation rate,* CRP* C reactive protein

Significant *p* value if <0.05*

The serum uric acid levels were significantly positively correlated with age, duration of Psoriasis, duration of PsA, BMI, CRP, ESR, DAPSA, and PASI score (Table 3). Significant p value if < 0.05*

**Table 3:** Correlation between serum uric acid and different variables

Variable	r	*p*
Age	0.25	0.002*
Duration of Psoriasis	0.25	0.002*
Duration of PsA	0.45	0.0001*
BMI	0.19	0.019*
CRP	0.44	0.0001*
ESR	0.43	0.0001*
DAPSA	0.39	0.0001*
PASI	0.32	0.0001*

*BMI* body mass index,* CRP* C reactive protein, *ESR* erythrocyte sedimentation rate, *DAPSA Disease Activity Index for Psoriatic Arthritis, PASI *Psoriasis Area and Severity Index

Significant *p* value if <0.05*

By using the univariate and multivariate logistic regression analysis to detect the variables associated with hyperuricemia in PsA patients, we found that male sex, BMI, and increased disease activity were independent predictors of hyperuricemia in PsA patients (Table 4).

**Table 4:** Variables associated with hyperuricemia in PsA patients

	Univariate		multivariate	
	OR (95% CI)	*P*	OR (95% CI)	*p*
Age	0.911(0.869–0.954)	0.0001*	1.006(0.946–1.069)	0.855
Sex	52.136(6.875–395.37)	0.0001*	150.32(11.97–1888.15)	0.0001*
BMI	0.622(0.512–0.757)	0.0001*	0.352(0.2–0.619)	0.0001*
PASI	0.89(0.78–1.02)	0.083	0.962(0.945–1.435)	0.848
DAPSA	0.865(0.819–0.913)	0.0001*	0.797(0.711–0.894)	0.0001*
Duration of PsA	0.958(0.94–0.976)	0.0001*	0.984(0.961–1.009)	0.205

*BMI* body mass index, *PASI* Psoriasis Area and Severity Index, *DAPSA Disease Activity Index for Psoriatic Arthritis,* *PsA* psoriatic arthritis

Significant *p* value if < 0.05*

## Discussion

In this study, we investigated the characteristics of PsA patients with hyperuricemia, compared the hyperuricemic and normouricemic PsA patients, and assessed the effect of hyperuricemia on clinical presentation, disease activity, disease severity, and associated comorbidities. We found that PsA patients with hyperuricemia had higher age, BMI, DAPSA score, ESR, CRP, serum cholesterol and triglycerides, and longer duration of psoriasis and psoriatic arthritis than PsA patients with normal serum uric acid levels. while there was no significant difference regarding gender, PsA articular subtype, PASI score, and treatment received. PsA patients with hyperuricemia had more associated comorbidities (such as hypertension, diabetes mellitus hypercholesterolemia, and hypertriglyceridemia) than PsA patients with normal uric acid as we found that there were significant increase in percentage of incidence of comorbidity in hyperurecemic group (63.2%) in comparison with those in normourecemic group (20.3%), this could be explained as serum uric acid is a marker of dysmetabolism, and detection of increased serum uric acid levels may enable earlier implementation of preventive measures for metabolic disturbances [15].

AlJohani R et al., [6] Reported that 31.2% of PSA patients had hyperuricemia. PSA patients with hyperuricemia had higher PASI score, ESR, CRP, and longer duration of PSA than PSA patients with normal serum uric acid, however, no significant difference regarding tender or swollen joints. Also, hyperuricemia had been associated with more comorbidities such as hypertension, diabetes, obesity, and kidney disease. In PsA patients, kidney stones, obesity, and disease duration were independent predictors of hyperuricemia.

Another study revealed that hyperuricemia was found in 30% of PSA patients while the prevalence of gout was 6.2%. In PSA patients with hyperuricemia had an older age, higher BMI, more polyarticular joint involvement, more peripheral joint destruction, poorer response to treatment of PSA, and more cardiovascular and renal disease when compared to PSA patients with normo-uricemia [8].

In a Chinese study, the prevalence of hyperuricemia was 30.6% in PSA patients, while in a Japanese study, the prevalence of hyperuricemia was 22%. The difference in the results regarding the prevalence of hyperuricemia may be attributed to the difference in serum uric acid level thresholds used in these studies. Furthermore, some studies measured serum acid levels at a single time point [7, 16].

Chu BBR et al.,[17] found no statistically significant difference regarding disease activity, tender or swollen joints, and CRP between PsA patients with and without hyperuricemia. This finding is confirmed by other authors [7, 18, 19].

Furthermore, a previous study demonstrated the correlation between hyperuricemia in psoriatic arthritis and obesity [20].

Numerous studies have shown that people with PsA are more likely than the general population to develop cardiovascular events and may even have a higher risk than those with only psoriasis [21–23].

In PsA patients, Pro-inflammatory cytokine production, especially interleukin-17 (IL-17), affects liver metabolism and may contribute to non-alcoholic fatty liver disease. Hepatocytes produce more uric acid when adenosine triphosphate synthesis declines due to hepatic steatosis [24]. This could explain increased uric acid levels in our patients as disease activity is one of the risk factors for hyperuricemia.

Serum uric acid has a positive association with inflammatory markers such as TNF-α, CRP, and IL-6 which indicates that uric acid has a role in systemic inflammation. Also, chronic inflammatory disorders associated with prolonged disease duration in PsA patients might encourage the release of proinflammatory cytokines which have a positive association with elevated levels of uric acid [25].

In this study, male sex, BMI, and increased disease activity were independent predictors of hyperuricemia in PsA patients. PASI score (psoriasis severity), age and duration of PsA weren’t predictors for hyperuricemia, in contrast to the systematic review which reveals a strong association between PsO and PsA with an increased incidence of HUA and gout [5].

There were contradictory results regarding the relation between severity of skin psoriasis and hyperuricemia, several studies found an association between skin psoriasis and hyperuricemia [9, 19, 26], while other studies found no correlation between serum uric acid and psoriasis severity [18, 27]. This discrepancy in outcomes could be attributed to differences in sample size and might be attributed to the fact that most of our cases didn’t have severe skin affection.

Monosodium urate crystals may have a detrimental impact on PsA, often known as psout. In clinical practice, it’s necessary to rule out gout attacks when a PsA flares up. Furthermore, recognizing hyperuricemic PsA could result in even more individualized treatment [2].

The main strength of our study is that this is a multi-centric study with a large number of patients included, and we avoided the confounding factors by measuring the mean values of at least 4 visits through the 12-month duration. But still some limitations as the retrospective nature of the study limits its power, in the same way, the prevalence of hyperuricemia and gout in PSA wasn’t measured. A prospective, longitudinal study is needed to clarify the genuine relationship between PSA and hyperuricemia.

## Conclusion

Psoriatic arthritis patients with hyperuricemia have higher age, BMI, disease activity, and more associated comorbidities. In PsA patients, hyperuricemia was associated with male sex, BMI, and increased disease activity, but not associated with PASI score.

## Supplementary Information

Below is the link to the electronic supplementary material.

ESM 1 (DOCX 34.1 KB)

## Data Availability

The data will be available upon reasonable request.
